# Endangered plant-parrot mutualisms: seed tolerance to predation makes parrots pervasive dispersers of the Parana pine

**DOI:** 10.1038/srep31709

**Published:** 2016-08-22

**Authors:** José L. Tella, Francisco V. Dénes, Viviane Zulian, Nêmora P. Prestes, Jaime Martínez, Guillermo Blanco, Fernando Hiraldo

**Affiliations:** 1 Department of Conservation Biology, Estación Biológica de Doñana, CSIC. Américo Vespucio s/n, E-41092 Sevilla, Spain; 2 Departamento de Ecologia, Universidade Federal do Rio Grande do Sul, Av. Bento Gonçalves, 9500 Porto Alegre, Rio Grande do Sul, 91540-000, Brazil; 3 Instituto de Ciências Biológicas, Universidade de Passo Fundo. BR 285, Bairro São José, Passo Fundo/RS, CEP: 99052-900, Brazil; 4 Department of Evolutionary Ecology, Museo Nacional de Ciencias Naturales, CSIC. José Gutiérrez Abascal 2, 28006 Madrid, Spain

## Abstract

Parrots are largely considered plant antagonists as they usually destroy the seeds they feed on. However, there is evidence that parrots may also act as seed dispersers. We evaluated the dual role of parrots as predators and dispersers of the Critically Endangered Parana pine (*Araucaria angustifolia*). Eight of nine parrot species predated seeds from 48% of 526 Parana pines surveyed. Observations of the commonest parrot indicated that 22.5% of the picked seeds were dispersed by carrying them in their beaks. Another five parrot species dispersed seeds, at an estimated average distance of c. 250 m. Dispersal distances did not differ from those observed in jays, considered the main avian dispersers. Contrary to jays, parrots often dropped partially eaten seeds. Most of these seeds were handled by parrots, and the proportion of partially eaten seeds that germinated was higher than that of undamaged seeds. This may be explained by a predator satiation effect, suggesting that the large seeds of the Parana pine evolved to attract consumers for dispersal. This represents a thus far overlooked key plant-parrot mutualism, in which both components are threatened with extinction. The interaction is becoming locally extinct long before the global extinction of the species involved.

Plant-animal mutualistic interactions are increasingly threatened by anthropogenic environmental degradation^1^. In particular, the loss of seed dispersal mutualisms has been mainly attributed to the extinction and decline of large endozoochrous dispersers^2^,^3^. Despite the fact that most recognized seed predators can also act as effective seed dispersers of their food plants^4^,^5^, their role in ecosystem integrity and resilience has been much less investigated. However, some seed consumers serve a pervasive dispersal function due to their widespread distribution, high mobility, large size and comparatively large abundance and biomass in communities of fruit and seed eaters^6^,^7^. Among birds traditionally considered as pure seed predators, parrots (Psittaciformes) have been recently highlighted as potentially important seed dispersers of their food plants by carrying seeds in their beaks or feet^8^,^9^. This type of external dispersal generally involves relatively large seeds that are moved long distances in flight and dispersed when the seed is discarded after the pulp is eaten, or when parrots spit out or accidentally drop the seeds^8^,^9^,^10^. However, there is no detailed information about the influence of external seed dispersal by parrots on seed germination, which precludes a proper evaluation of the function of parrots as seed dispersers.

Fruits handled by seed eaters are often de-fleshed with seeds partially consumed or damaged without affecting seed viability if the embryo is left unharmed and if the greater part of the reserves used for seedling establishment is not consumed^11^,^12^,^13^,^14^. Indeed, some species retain the ability to germinate and establish saplings even from seed tissues with damaged embryonic axes^15^. In addition, partial seed consumption and seed handling, such as scarification, scratching and scarring can enhance germination by promoting moisture entry and increased water intake through slits or pecking sites on the outer protective, often very hard, seed coat and other physical barriers^13^,^14^,^16^. The typical wasteful and mobile foraging of parrots generally implies that a large number of seeds of a variety of plants are dropped intact or only partially damaged under the canopy of fruiting plants^8^,^17^ or after being moved to distant perches for handling^8^,^9^,^10^. There is, however, a general lack of information on the impact of parrot partial consumption and other damage on the germination of large seeds, which can represent a primary component of their diets (e.g. ref. 8).

Parrots have been observed massively feeding on the large seeds of the Parana pine (*Araucaria angustifolia*)^18^,^19^,^20^,^21^. This is a Critically Endangered species, mostly distributed through southern and southeastern Brazil^22^. The original distribution is believed to have declined by more than 97% in the last century due to massive exploitation for timber, generating a mosaic of small forest patches^22^,^23^. Both dispersal and seed consumption can influence patterns of colonization of Parana pine in the current landscape mosaic^24^. The Parana pine produces a big (5–8 cm) and highly energetic seed (weighting c. 7 g), each female producing on average 13–20 cones with 80–90 seeds per cone after 20–24 months of maturation^25^. Seed maturation and seed fall occur from March to June^25^, providing a key food resource for a variety of mammal and bird species acting as seed predators and dispersers^21^. Among them, three species of parrots have been considered important predators consuming large quantities of seeds of this endangered species, assuming they fully destroy the seeds, and occasionally dispersing them^21^.

In this study, we evaluated whether parrots act as legitimate seed dispersers promoting recruitment of Parana pines even after partially consuming the seeds. This relies on the hypothesis that large seeds, such as those of Parana pine, can tolerate partial predation and can therefore benefit from attracting consumers for dispersal^11^,^26^,^27^. To test this hypothesis, we first assessed the extent of seed-parrot interactions by a) estimating to what extent each parrot species exploited this resource, recording signs of seed predation by parrots under 526 seed-producing Parana pines selected in four distant study areas, and b) estimating the relative abundance of each parrot species in the wild through road-side surveys. The same kind of information was collected from jays (Passeriformes, Corvidae), considered as the main avian dispersers of Parana pine seeds^21^, for comparative purposes. Second, by conducting detailed observations of foraging individuals, we evaluated the occurrence of external seed dispersal (i.e., individuals carrying seeds in their beaks), and quantified seed dispersal rates and dispersal distances. Third, we looked for germinating seeds, recording whether they were undamaged or partially eaten by parrots, and assessed whether germination rates differed between undamaged and partially eaten seeds. We finally discuss the role of parrots in this animal-plant mutualism system where both components are threatened by extinction.

## Methods

### Study areas and species

**Figure 1:**
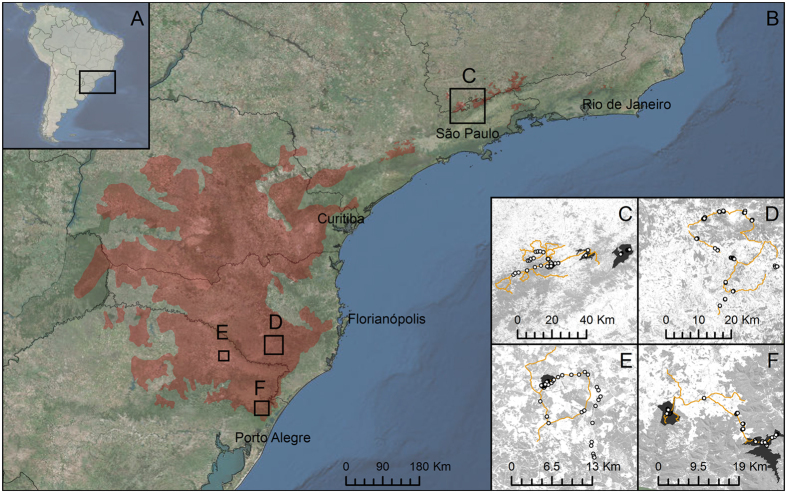
Study area and sampling sites. Location of the study region in South Brazil (**A**) the broad-scale distribution (dark red) of Parana pine forests (**B**) and the four selected study areas (**C–F**) showing the surveyed Parana pine females (white dots, note many of them overlap), road-side surveys (yellow lines), the patchy distribution of any kind of forests (light grey), and protected areas (dark grey). Maps were generated using ArcGIS v.10.2.1. and Land**s**at-4 images available at http://glovis.usgs.gov/.

Based on long-term knowledge acquired by the research team on the ecology of parrots inhabiting Parana pine forests^18^,^19^,^28^ and field work conducted on 1–12 May 2015, we selected four large, distantly located study areas to cover spatial and habitat variability and the entire distribution of the parrot species that feed on Parana pine seeds (Fig. 1). These study areas were: (Fig. 1C) Serra da Mantiqueira region, Minas Gerais and São Paulo states (Municipalities of Gonçalves [MG], Sapucaí-Mirim [MG], Camanducaia [MG], Campos do Jordão [SP] and São Bento do Sapucaí [SP]). Protected areas visited included Parque Estadual de Campo-do-Jordão and Monumento Natural Estadual da Pedra do Baú; (Fig. 1D) Serra Catarinense region, Municipalities of Painel and Urupema, Santa Catarina state; (Fig. 1E) Muitos Capões municipality, Rio Grande do Sul state, including the protected area of Estação Ecológica de Aracuri-Esmeralda; and (Fig. 1F) São Francisco de Paula municipality, Rio Grande do Sul, including the protected areas Floresta Nacional de São Francisco de Paula and Centro de Pesquisas e Conservação da Natureza Pró-Mata (PUC-RS).

Three species of parrots, the red-spectacled amazon (*Amazona pretrei*), the vinaceus amazon (*Amazona vinacea*) and the maroon-bellied conure (*Pyrrhura frontalis*), as well as two species of jays, the azure jay (*Cyanocorax caeruleus*) and the plush-crested jay (*Cyanocorax chrysops*), have been recorded consuming and eventually dispersing Parana pine seeds (see ref. 21 for the most updated review). Through this study, we recorded six additional species of parrots and another jay species inhabiting fragments of Parana pine forests: yellow-chevroned parakeet (*Brotogeris chiriri*), peach-fronted parakeet (*Eupsittula aurea*), monk parakeet (*Myiopsitta monachus*), white-eyed parakeet (*Psittacara leucophthalmus*), pileated parrot (*Pionopsitta pileata*), scaly-headed parrot (*Pionus maximiliani*), and curl-crested jay (*Cyanocorax cristatellus*).

### Seed predation

Within the four study areas, we selected 526 mature female trees for assessing their use by parrots and jays between May 11 and June 6, 2015, thus covering the end of the seed production period of Parana pine^25^. Selected trees (Fig. 1C–F) covered a wide environmental gradient, from 802 to 1820 m a.s.l., distributed throughout forest patches differing in size and connectivity and including isolated trees in grasslands, pastures and cultivated landscapes (see ref. 10 for a similar approach).

Selected trees were spaced sufficiently from the closest mature female tree to avoid confusion when assigning fallen seeds to parental trees. However, there is the possibility that some seeds did not fall from the sampled tree but were instead dispersed to it by parrots. Conversely, some seeds from the selected tree could have been dispersed away by parrots without leaving evidence of parrot predation. Our results on the percentage of trees whose seeds were predated by parrots must thus be interpreted as approximate rather than exact figures. Under each tree, 2–3 persons inspected the ground within a radius of 10–20 m (depending on the canopy size of the tree) for 5–10 min (i.e. the time needed for full inspection), looking for fallen seeds. From the first 30 seeds found (or less if there were few seeds under the tree), we recorded how many seeds were undamaged and how many were predated (following ref. 10).

The identity of seed predator species was reasonably recorded attending to the different tooth or beak marks found on predated seeds and the way they were predated^10^,^29^, which even allowed the identification of different parrot species. This was facilitated by the experience accumulated during two decades by two of the authors (NPP and JM), studying the feeding ecology of parrots in Parana pine forests and in captivity^18^,^19^,^20^, and by the recent experience of FH and JLT studying seed predation in a sister species, the monkey puzzle tree (*Araucaria araucana*)^10^. Nonetheless, the fieldwork team was first trained by observing foraging parrots with binoculars and telescopes and later examining the predated seeds they dropped to confirm differences among species. Direct observations were complemented with infra-red camera traps to identify both diurnal and nocturnal seed predators, within an accompanying work aimed to study Parana pine seed predation by native and exotic species (F.V. Dénes, V. Zulian, N.P. Prestes, J. Martinez, J.L. Tella & F. Hiraldo, in prep). Briefly, parrots hold the seed with a foot to open the seed coat with the upper mandible, leaving the marks of the lower mandible in the opposite side of the seed. Some pictures are provided in Supplementary Information to illustrate differences among seeds predated by different species. The large-sized parrots (*Amazona pretrei* and *A. vinacea*) largely open the seed from the distal to the basal section of the seed, with slight differences in the way the seed coat is destroyed and with a larger damage generally caused by *A. vinacea* than by *A. pretrei*. Both species however drop some partially eaten seeds. The mostly disjunct distribution of these species in our study areas also helped to identify the *Amazona* species that predated seeds; in a few cases, however, we could not assign species identity and thus we recorded seeds as predated by *Amazona* sp. The medium-sized *P. maximiliani* opens the middle area of the seed, causing less damage than *Amazona* species. The small-sized *P. frontalis* just opens a small section of the distal area of the seed coat, from where they often extract the whole seed. The rest of small-sized parrots (*M. monachus, P. leucophthalmus and E. aurea*) open the seed coat in a similar way that *P. frontalis*. Seed predation by these species was identified by their disjunct distribution across the forest fragments studied and direct observations. Seed predation by jays was distinctive, since they perforate with the beak a full side of the seed with repeated blows, not leaving the typical marks of the lower mandible of parrots in the opposite side of the seed. As for small-sized parrots, the identity of jay species was inferred from their disjunct distribution across forest patches and direct observations. Seeds predated by mammals were clearly distinguishable from those predated by birds, and were often accompanied by fresh tracks and feces. For example, small rodents leave small teeth marks (S1E). In some cases the seed was partially eaten by a parrot and latter nibbled by a small rodent when dropped to the ground. Larger mammals cause larger damage on seeds. For example, European hares (*Lepus europaeus*) nibble all but the basal part of the seed, and the largest species such as deer, wild boar (*Sus scrofa*) and monkeys chew the whole seed leaving distinctive teeth marks. From these observations, we estimated the proportion of selected trees in which each parrot and jay species fed on seeds.

### Abundance of avian seed predators

We relied on roadside surveys^8^,^30^ to estimate the relative abundance of each parrot and jay species. We travelled 575 km of mostly unpaved roads throughout the four study areas (Fig. 1), driving a car at low speed (20–40 km/h) with three observers looking for parrots and jays. The distance to the first detection of each bird or group of birds was obtained through a laser rangefinder (range of measurements: 10–1300 m). Due to the relatively low number of contacts for most species, given that they often grouped in large flocks, we were not able to calculate species-specific detection probabilities for obtaining densities through distance modeling^31^. Thus, we relied on relative abundances of each species expressed as the number of individuals detected per km surveyed^8^,^30^. Nonetheless, our previous studies showed that relative abundances were strongly correlated (Spearman correlation, r_s_ > 0.9) to densities in different parrot communities (ref. 8, Denes, Tella & Hiraldo unpub. data).

### Seed dispersal

Recording seed dispersal events by parrots is difficult given the challenges of observing in detail their foraging behavior in forest habitats^9^,^10^ and finding foraging flocks given their aggregation and patchy distribution in the landscape^8^,^30^. Our roadside surveys allowed increasing the encounters with foraging flocks. Each time we saw or heard parrots and jays during the transect surveys, we attempted to locate them and verify whether they were foraging on Parana pine trees (additional individuals observed during foraging observations were not considered in relative abundance estimates). In these cases, one observer watched the foraging birds with a 20–50x telescope, while a second observer recorded data, and a third visually followed any bird that left the tree carrying a seed in its beak. When dispersal events were observed (i.e., seeds were transported from the mother tree to another site), we measured with a laser rangefinder the distance from the external border of the canopy to the point where the bird perched for handling the seed. In some cases the seed-dispersing bird flew behind the forest canopy and thus a minimum dispersal distance was measured to the point where the bird was out of sight. In a few instances, we found seeds under perches where parrots or jays were consuming them, and estimated the minimum dispersal distance as the distance to the closest fruiting female Parana pine tree. The landscape and forest structure did not allow us to record detailed foraging behavior most of the time. In two instances, however, we could observe large foraging groups of parrots from advantageous points, so we were able to record in detail the proportion of seeds that were consumed in the parental tree or that were dispersed to distant perches.

### Seed germination

We looked for germinating seeds below and around the 526 selected trees, recording whether germinating seeds were undamaged or partially eaten by any seed predator species. Given the relative small sample sizes, we pooled for analyses all seeds partially eaten by parrots.

We attempted to compare the proportion of undamaged/partially eaten germinating seeds with the availability of undamaged and partially eaten seeds, to assess whether partial predation affects germination success. Unfortunately, when designing the fieldwork we could not know to what extent partial predation can preclude germination. In other words, we lacked a clear *a priori* definition of partially eaten seed. Therefore, during the sampling of seeds under selected tress (see above) we only scored seeds as predated (pooling fully and partially predated seeds) or not (undamaged seeds). It was not until finishing the fieldwork when we learned that seeds with predation affecting <1/3 of the distal part of the seed (which often does not damage the embryo) still have the potential to germinate. Therefore, and after our fieldwork experience, we conservatively estimated that <5% of the seeds recorded as predated actually suffered partial predation affecting to <1/3 of the seed. We applied this proportion (5%) to the number of predated seeds recorded under selected trees to obtain a null expectation to be statistically compared with the proportion of undamaged/partially eaten seeds found germinating.

### Statistical analyses

Proportions were compared through Chi-squared tests. The proportion of trees with seeds predated by each parrot species was correlated to the relative abundance of parrot species through Spearman correlations. Because our dispersal data were often right-censored (i.e., we recorded many birds flying with a seed in the bill until they were out of sight, and measured the distance to the last point of observation, henceforth the minimum dispersal distance), we relied on failure-time analysis for estimating actual mean dispersal distances^32^,^33^. We employed an adaptation of Kaplan-Meier (or Product-Limit) estimators for survival functions^34^ to estimate dispersal functions, *D*(*d*), which inform the probability that a dispersal event will occur at a given distance. The Kaplan-Meier estimator provides an efficient means of estimating the survival (in our case, dispersal) function for right-censored data such as our dispersal dataset, in which both observed (exact distances) and unobserved (minimum distances) events occur. The Kaplan-Meier estimate of *D*(*d*) corresponds to the non-parametric MLE estimate of *D*(*d*), and is a step function with jumps at the observed event distances. The size of these jumps depends not only on the number of events observed at each event distance *d*_*i*_, but also on the pattern of the censored observations before *d*_*i*_. We estimated *D*(*d*) for jays and parrots separately, and tested for significant differences in dispersal distances for each group based on a log-rank test^34^. Mean and median dispersal distances were obtained from the estimated functions: the former is the integral of the dispersal curve, and the latter the intersection of the curve with a horizontal line drawn at 0.5^35^. We used the package *survival*^35^ in R^36^ to estimate dispersal functions and perform significance tests.

### Ethical statement

This work did not evolve experiments on live vertebrates that would require their approval.

## Results

### Seed predation and predator abundances

The percentage of surveyed female Parana pines (n = 526) in which we recorded seed predation by parrots greatly varied among parrot species (Chi-squared test, χ^2^_8_ = 426, p < 0.0001; Table 1). Seed predation by four species (*A. pretrei, A. vinacea, P. maximiliani* and *P. frontalis*) was recorded in ca. 8–20% of Parana pines, whereas predation by other species was much lower (<1.5%), and null in the case of *B. chiriri* (Table 1). In the case of *P. pileata*, the consumption of Parana pine seeds was only inferred from observations of birds mixed with foraging flocks of *P. frontalis.* The lowest percentages of trees with signals of seed predation corresponded to parrot species with marginal distributions in the northern (*B. chiriri, E. aurea, P. leucophthalmus*) and southern (*M. monachus*) range of the Parana pine (which we only recorded in study areas C and E-F, respectively), while *P. pileata* was only rarely recorded in study areas D and E (Fig. 1). Overall, seed predation by at least one parrot species was recorded in 48% of the trees while seed predation by jays only affected to 21.10% of the trees (Yates’ Chi-squared test, χ^2^_1_ = 81.37, p < 0.0001; Table 1).

**Figure 2:**
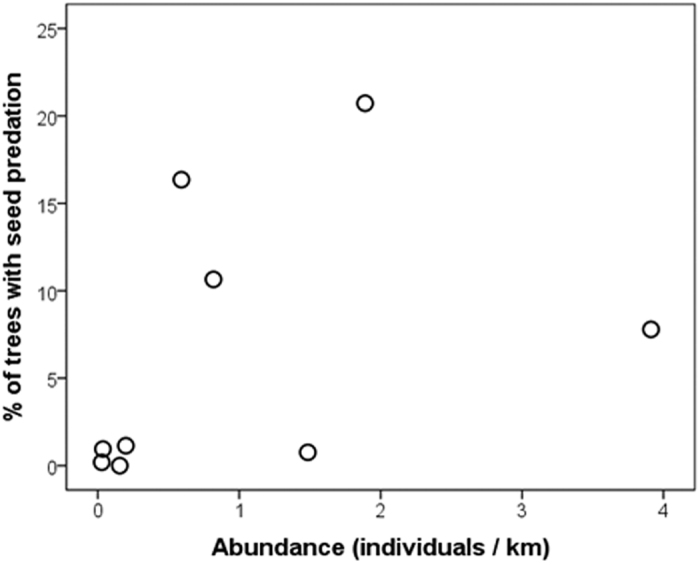
Relationship between the percentage of Parana pine trees with seeds predated by each parrot species and their relative abundances in the wild.

Parrot species greatly differed in their relative abundances in the wild (ranging from 0.036 for *P. pileata* to 3.91 individuals/km for *A. pretrei*), and overall parrot abundance was 40 times higher than overall abundance of jays (Table 1). The percentage of trees with signs of seed predation by each parrot species increased with their relative abundances in the wild (Fig. 2), although the correlation was only marginally significant (Spearman correlation, r_s_ = 0.62, p = 0.077, n = 9 species).

### Seed dispersal

**Figure 3:**
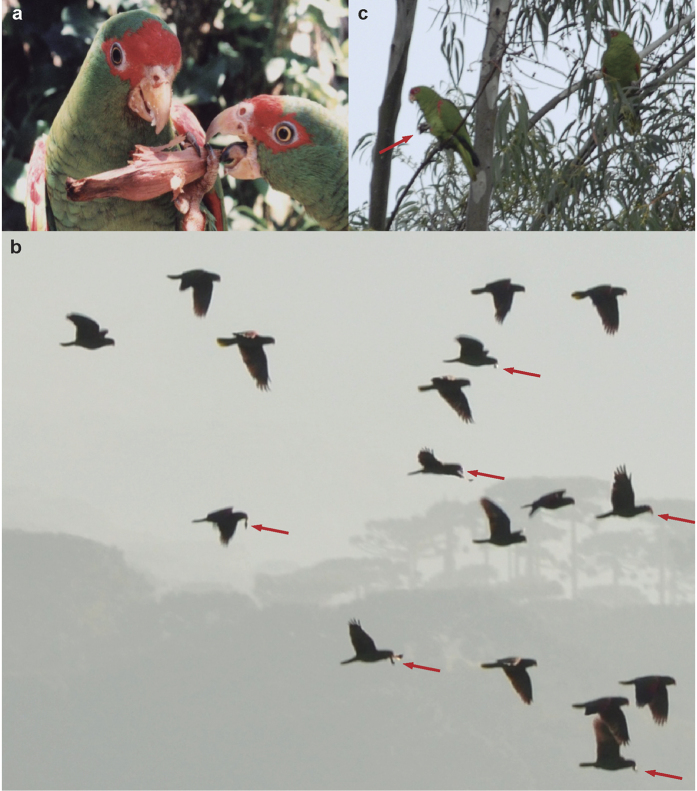
Seed dispersal process. Red-spectacled amazons (*Amazona pretrei*) pick up seeds from Parana pines (**a**) and often disperse the seeds carrying them in the bill (**b**) to distant perches to be handled and consumed (**c**). In c, one amazon perched on a distant *Eucaliptus* tree and dropped the partially-eaten seed. Photographs taken by N.P. Prestes (A), J. Martinez (B), and J.L.Tella (C).

We were able to quantify seed dispersal rates in two instances (25 and 29 May) when we could observe, from advantageous points, large flocks (ca. 600 and 100 individuals, respectively) of *A. pretrei* foraging on Parana pine seeds in study area D. Of 89 seeds extracted from female cones by different individuals (Fig. 3a), 69 were consumed in the same parental tree while 20 were dispersed in flight just after being extracted (Fig. 3b) for handling on distant perches (Fig. 3c). This rendered a 22.47% dispersal rate. Dispersal rates were similar in the first (20.34%, n = 59) and second (26.66%, n = 30) observation instances, which lasted 38 and 45 min, respectively (i.e., time from when the foraging flocks were encountered until they moved to a distant forest patch).

**Figure 4:**
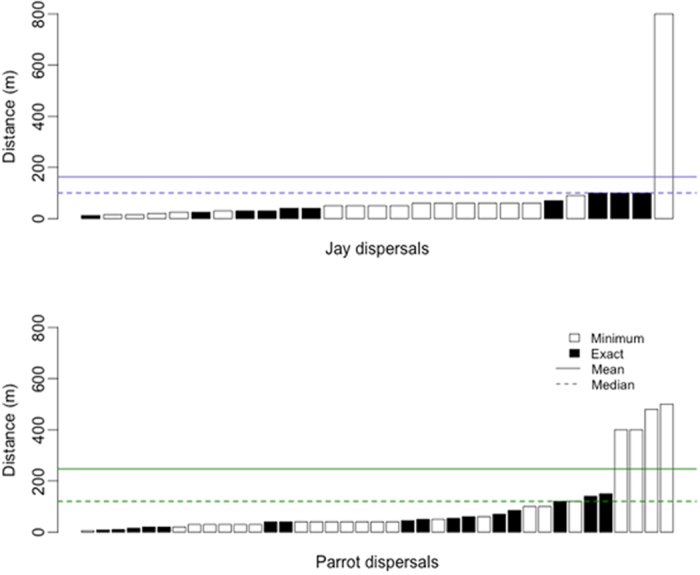
Parana pine seed dispersals by parrots and jays. Each bar represents a seed dispersal event (white bars: minimum distances, black bars: exact distances). Solid and dashed lines indicate, respectively, the estimates of mean and median dispersal distances from the dispersal functions for each group of species.

**Figure 5:**
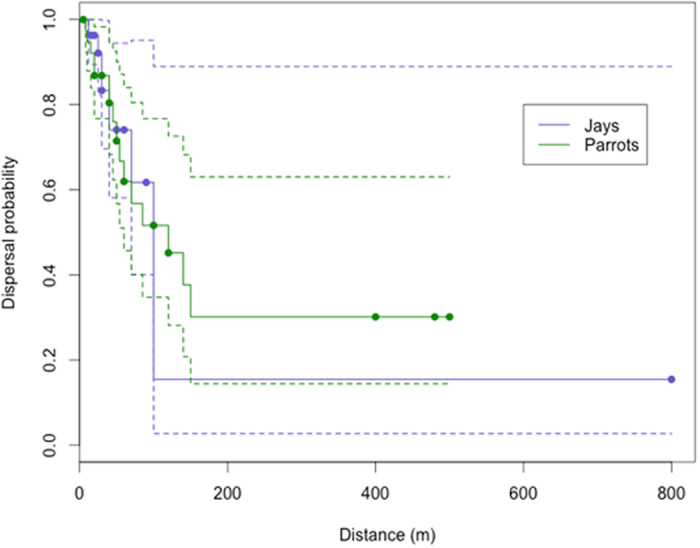
Kaplan-Meier estimates of dispersal functions for jays and parrots. Dashed lines show 95% confidence bounds, and solid circles indicate censored (minimum distance) observations.

Additional seed dispersal events were recorded during roadside surveys and visits to the 526 female trees, totaling 39 seed dispersions by parrots in which we were able to measure the exact or minimum dispersal distances. These ranged from 5 m to a minimum of 500 m (Fig. 4). Most dispersal events recorded were of foraging *A. pretrei* (n = 33, distances: 8–500 m), with additional cases of *A. vinacea* (n = 2, distances: 5 m and 120 m), *P. frontalis* (n = 1, 30 m), *P. maximiliani* (n = 1, 45 m), *M. monachus* (n = 1, 30 m), and *E. aurea* (n = 1, 20 m). We also recorded seed dispersal by Azure Jays *Cyanocorax caeruleus* (n = 26, 12–800 m) and Curl-crested Jays *Cyanocorax cristatellus* (n = 1, 12 m). Dispersal functions did not differ significantly between parrots and jays (χ^2^_1_ = 0, p = 0.913; Fig. 5). The estimates of mean (*μ*) and median (*m*) dispersal distances were: *μ*_parrots_ = 247 m (SE = 63.4), *m*_parrots_ = 120 m, *μ*_jays_ = 163 m (SE = 77.7) and *m*_jays_ = 100 m (Fig. 4). Among other bird species, we only saw seed dispersal by one Golden-winged Cacique *Cacicus chrysopterus* (distance 30 m).

### Seed germination

**Figure 6:**
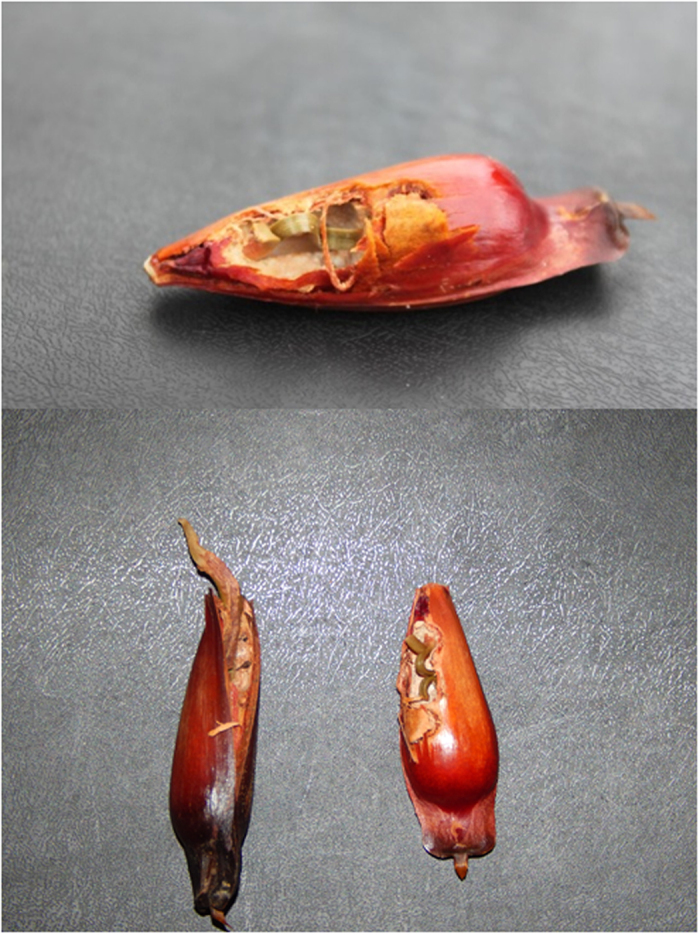
Parana pine seeds germinating after partial consumption by parrots. Photographs taken by F. Hiraldo and F.V. Dénes.

Of 6789 seeds examined under the 526 surveyed Parana pine female trees, 2058 (30.31%) were undamaged by predators and 4731 (67.69%) were totally or partially predated. Although we did not accurately count the number of partially eaten seeds, we estimated that their proportion with respect to fully eaten seeds was <5% (see Methods). Among the 125 seeds that were found germinating, only 32.8% of them were undamaged, while the rest (67.2%) were partially eaten by seed predators (Table 2). The large percentage of germinating seeds that were previously damaged by predators differed from that expected by chance when conservatively assuming a 1/20 ratio of partially eaten/fully eaten seeds (Chi-squared test, χ^2^_1_ = 365, p < 0.0001). Damage to seeds that germinated usually affected less than one-third of the distal part of seed, although in some instances the damage was much greater (Fig. 6). Damage was attributed to parrots (*P. frontalis, P. maximiliani, A. pretrei* and *A. vinacea*) in 22.4% of the cases and to rodents in 13.6% of the cases. In 29.6% of the cases, the extent of damage caused by the predator species could be not unambiguously determined, since the seed was initially pecked by parrots and then additionally damaged my mice once it fell to the ground. Only two germinating seeds were damaged by Lepidoptera larvae, and none of the germinating seeds were partially eaten by jays.

## Discussion

Eight of the nine parrot species present in the study areas were recorded consuming seeds of Parana pines, in contrast with previous information which only indicated seed predation by three parrot species (*A. pretrei, A.vinacea* and P. *frontalis*)^18^,^19^,^21^. The proportion of sampled female Parana pines with predated seeds under the canopy varied among parrot species, being highest for the four most abundant species. However, there was no strong correlation between predation rates (at the tree level) by parrot species and their relative abundances in the wild. This may be explained by differences in the contribution of Parana pine seeds to their winter diet, of which little is known. Parana pine seeds contribute significantly to the diets of *A. pretrei* and *A. vinacea*^18^,^19^,^37^, but may be much lower in other species. In addition, the patchy distribution of parrots across the Parana pine range could contribute to this weak correlation between predation rates and abundance.

By conducting detailed observations of focal *A. pretrei* individuals foraging in large flocks, we were able to confirm that this species disperses Parana pine seeds carrying them with the beak at an approximate rate of 22.5% of the handled seeds. Previously, Kindel^29^ considered dispersal of Parana pine seeds by this species as anecdotal, having recorded only one, albeit long-distance (ca. 1 km), dispersal event. Dispersal distances by parrots ranged from 5 to 500 m, with an estimated mean of 247 m. It is worth noting that dispersals performed by parrots were at least as distant as those performed by jays, whose role as dispersers of the Parana pine is well recognized^21^,^29^. In fact, the estimated dispersal functions for both groups did not differ significantly, indicating that parrots and jays are similarly capable of dispersing seeds (in terms of distance) away from parent trees. However, jays seem to play a secondary role compared to parrots, given their much lower abundances in the wild and the lower percentage of trees they visited for picking seeds. Jays may even be less efficient dispersers since they usually eat the whole endosperm and embryo^29^. In fact, we found seeds clearly predated by jays under 21% of the 526 surveyed female trees, and all of those seeds were fully predated. On the other hand, jays can still accidentally drop undamaged seeds, and there are anecdotal records of them hiding seeds underground or in the undergrowth, a behavior that requires further investigation^29^.

Among parrots, we were only able to obtain dispersal rates from foraging *A. pretrei*, probably due to its large abundance during the sampling period. However, we also observed several seed dispersions conducted by *P. frontalis* and *A. vinacea*, which were previously recorded performing dispersal events of up to 20 m and 100 m, respectively^37^. We also observed seed dispersal by another three parrot species, which had not been previously recorded as dispersers (or as predators) of Parana pine seeds^21^. This illustrates the difficulty of observing in detail foraging parrots in the wild, and as a result they may have been largely overlooked as seed dispersers^8^,^9^,^10^,^38^. In fact, we feel our survey underestimated the dispersal capacity of *P. frontalis* compared to Amazon parrots, given that its smaller size and different behavior make it difficult to observe their foraging activities within the tree foliage in detail. However, our single observation together with previous records^37^ suggests that this species moves seeds to much shorter distances than the larger *Amazona* parrots.

Secondary dispersal can be performed by a variety of cache-hoarding rodents and other vertebrates, as stated previously for the Parana pine^21^ and other plant species with large seeds^8^. Our work confirms previous suggestions^21^,^37^ and shows that parrots are important in dispersing Parana pine seeds far from the crown of mother plants. In one instance, we observed one *P. maximiliani* dropping an undamaged seed after dispersing it 45 m and, although not properly quantified, we often saw parrots dropping seeds after only minor damaged or even dropping undamaged seeds in flight (Fig. 3). Importantly, as recorded for the congeneric monkey puzzle tree *Araucaria araucana*^10^ and other plant species^8^,^9^,^17^,^39^,^40^, parrots can act as primary dispersers by moving most seeds long distances (hundreds of meters), which can have a pervasive influence in the genetic structure of populations. Genetic mapping of all living trees showed the realized dispersal of seeds within a 7.2 ha plot of Parana pine forest, dispersal reaching 318 m with 50% of seeds being dispersed up to 133 m^41^. Given the relatively short movements of mice and medium-sized rodents^42^,^43^, parrots may play a key role on long-distance seed dispersal, thus facilitating the regeneration and connection of fragmented population nuclei of this endangered species. This may be particularly important today, when many small patches of Parana pine are embedded in a matrix of pastures and crops (ref. 23; Fig. 1C–F), which may be hostile to the movements (and thus seed dispersal) of forest rodents, but not for the long-flying movements of parrots.

Among the seeds found germinating under the canopy of the sampled trees, two-thirds were partially eaten by seed predators. Damage by parrots was found in almost 60% of the seeds in which unique predators were identified, with rodents showing a much lower proportion. Moreover, an important proportion of germinating seeds were partially eaten by both parrots and mice. These estimates are clearly conservative, because we only searched for germinating seeds under the canopy of mother plants, thus missing those dispersed and dropped by parrots in other trees (male Parana pines or other tree species) or outside the forest, and those cached by rodents. Partial consumption by parrots usually affected less than one-third of the upper distal part of the seed, generally without damaging the radicle and cotyledons. This indicates that seeds of Parana pine can successfully germinate after losing a variable, but not yet properly quantified, part of the storage tissue, as reported for other large-seeded species^11^,^12^,^14^,^26^,^27^,^44^. The fact that the proportion of partially eaten seeds that germinated was higher than that of undamaged seeds may suggest that partial seed consumption can enhance germination^37^. By partially removing the seed coat, parrots could be eliminating the main barrier to moisture while favouring subsequent water intake and seedling emergence^13^,^14^,^16^. In fact, laboratory experiments have shown that the scarification of Parana pine seeds (by cutting a small distal portion of the seed) reduces fungal infections and increases their germination success^45^, also promoting more rapid initial growth and vigour of seedlings^46^.

Our results indicate that parrots are legitimate dispersers of Parana pines, occasionally dispersing undamaged seeds but mostly dispersing partially eaten seeds with capacity to germinate, and that seed predation activity by parrots^21^ has been overestimated. Partial consumption of large seeds by animals has been mainly studied in oaks, whose acorns can tolerate partial predation to germinate and establish saplings^11^,^27^. This suggests that large seeds have evolved to attract seed consumers for seed dispersal^11^,^26^,^27^. The fact that seeds of the Parana pine have more reserves than those required for germination suggests a similar pattern of attraction of seed consumers that may also act as seed dispersers. Nonetheless, more research is needed to assess the effect of partial predation on seedling establishment and growth.

Finally, we are able to roughly approach the potential function of parrots as legitimate seed dispersers of the Parana pine, considering the two species (*A. pretrei* and *A. vinacea*) on which much more information on their ecology and population sizes is available. Parana pine seeds constitute most of their diet during winter^18^,^19^,^37^. Prestes *et al*.^20^ studied the daily seed intake of *A. pretrei* and *A. vinacea* in captivity during a 180-day period, finding that individuals can consume on average 10.05 kg and 10.52 kg of Parana pine seeds during the winter, respectively. Individuals of these two species discarded 28.6% and 20.95% of food as partially eaten seeds, respectively. Considering an average seed weight of 7 g^25^, a seed dispersal rate of 22.5% (this study), and the fact that the world winter population of *A. pretrei* and *A. vinacea* was estimated at 15,685 and 3,133 individuals, respectively, in 2014 (unpublished census coordinated by N. Prestes and J. Martinez), these two parrot species alone could be dispersing more than 1.6 million partially eaten seeds with potential to germinate per year. Even assuming a very low 1% germination success of these partially eaten, dispersed seeds, up to 16,000 new trees could grow annually far from their mother plants thanks to *Amazona* parrots.

These are rather simple estimations, not accounting for any other, albeit secondary, food sources or for the higher energy requirements of individuals in the wild than in captivity. However, they allow us to illustrate how important the, thus far, overlooked role of parrots may be for the dispersal and regeneration of Parana pine forests. Moreover, they illustrate how anthropogenic changes can threaten important species interactions to the point of extinction of the interaction before the actual disappearance of the species^1^. The harvesting of Parana pinecones for human consumption, while still in the tree crowns, together with seed predation by introduced exotic mammals (ref. 22 and 47; F.V. Dénes, V. Zulian, N.P. Prestes^3^, J. Martínez, J.L. Tella & F. Hiraldo, in prep.), is likely to impact not only the regeneration of this tree species but also the plant-parrot mutualistic interaction. IUCN lists *A. pretrei* as Vulnerable and *A. vinacea* as Endangered, due to their large population declines associated with the large-scale extirpation of the Critically Endangered Parana pine, but also as a consequence of illegal parrot poaching for the pet market^48^. *A. vinacea* is still widely distributed, although at low densities, throughout the range distribution of Parana pine. However, the much more abundant *A. pretrei* experienced a large range contraction^49^ and, as of two decades ago, nearly the entire world population concentrates during the winter in study area D (ref. 28; N. Prestes and J. Martinez, unpubl. data), probably because forest patches are not large enough in other regions to provide reliable food (i.e., Parana pine seed sources) throughout the winter. Therefore, an endangered plant-parrot mutualism has already disappeared at regional scales, long before the global extinction of the species involved. Moreover, a further decline of these and other parrot species acting as dispersers of Parana pines can hinder forest regeneration, eventually increasing the chance of both partners becoming extinct at regional or global scales.

This study supports recent evidence indicating that a variety of parrot species may act as key dispersers of a variety of plant species^8^,^9^,^10^,^17^,^38^,^39^,^40^. Given that nearly one third of the parrot species of the world are threatened with extinction due to anthropogenic habitat changes^50^, and also to overharvesting for the pet trade^51^, it is crucial to increase research efforts to identify and better understand parrot-plant mutualisms before many of them become locally or even globally extinct.

## Additional Information

**How to cite this article**: Tella, J. L. *et al*. Endangered plant-parrot mutualisms: seed tolerance to predation makes parrots pervasive dispersers of the Parana pine. *Sci. Rep.* **6**, 31709; doi: 10.1038/srep31709 (2016).

## Supplementary Material

Supplementary Information

## Figures and Tables

**Table 1:** Use of Parana pine trees by each bird species and their relative abundances.

Species		N	%	Area	Indiv.	RA
Red-spectacled amazon	*Amazona pretrei*	41	7.79	D, E	2252	3.912
Vinaceus amazon	*Amazona vinacea*	56	10.65	C, D, F	471	0.818
Amazon sp.	*Amazona sp.*	3	0.57	D, F	3	0.005
Yellow-chevroned parakeet	*Brotogeris chiriri*	0	0	C	89	0.155
Peach-fronted parakeet	*Eupsittula aurea*	1	0.19	C	16	0.028
Monk parakeet	*Myiopsitta monachus*	6	1.14	E, F	114	0.198
Pileated parrot	*Pionopsitta pileata*	5	0.95	D, F	21	0.036
Scaly-headed parrot	*Pionus maximiliani*	86	16.35	C, D, F	340	0.591
White-eyed parakeet	*Psittacara leucophthalmus*	4	0.76	C	855	1.485
Maroon-bellied conure	*Pyrrhura frontalis*	109	20.72	C, D, E, F	1088	1.890
TOTAL PARROTS		251	47.72		5346	9.297
Azure jay	*Cyanocorax caeruleus*	99	18.82	D, E, F	69	0.120
Plush-crested jay	*Cyanocorax chrysops*	4	0.76	C, E, F	1	0.001
Curl-crested jay	*Cyanocorax cristatellus*	8	1.52	C	61	0.106
TOTAL JAYS		111	21.10		131	0.228

Number (N) and percentage (%) of female trees (n = 526) with recorded seed predation by parrots and jays, with the number of individuals and relative abundance (RA = individuals/km) of species observed through road-survey counts (575 km), and the areas where they were recorded (see Fig. 1).

**Table 2:** Number and percentage of germinating seeds (n = 125) that were undamaged or partially consumed by different seed predators.

Seeds	Species	N	%
Partially predated	Parrots	28	22,40
Partially predated	Rodents	17	13,60
Partially predated	Parrots+rodents	37	29,60
Partially predated	Lepidoptera	2	1,60
Undamaged		41	32,80
