# Supplementary material for: Endangered plant-parrot mutualisms: seed tolerance to predation makes parrots pervasive dispersers of the Parana pine
Source: Sci Rep. 2016 Aug 22;6:31709. doi: 10.1038/srep31709 (PMC4992845; doi:10.1038/srep31709)
Supplement: Supplementary Information [file srep31709-s1.pdf]

Endangered plant-parrot mutualisms: seed tolerance to predation makes parrots pervasive dispersers of the Parana pine

José L. Tella, Francisco V. Dénes, Viviane Zulian, Nêmora P. Prestes, Jaime Martínez, Guillermo Blanco & Fernando Hiraldo

Supplementary Information

Figure S1. Some examples of Parana pine seeds predated by different bird and mammal species:

S1a) Vinaceus amazon *Amazona vinacea*.

S1b) Red-spectacled amazon *Amazona pretrei*.

S1c) Scaly-headed parrot *Pionus maximiliani*.

S1d) Maroon-bellied conure *Pyrrhura frontalis*.

S1e) Small rodents (Family Cricetidae).

S1f) In some cases parrots, in this case maroon-bellied conure, opened the distal part of the seed (arrow d), and later on some small rodent nibbled the central area of the seed (arrow e). Damage by parrots and mice sometimes overlapped to the point of making difficult to identify which species contributed more to partial predation of the seed.

S1g) European hare *Lepus europaeus*.

S1h) Red brocket *Mazama americana*.

S1i) Wild boar *Sus scrofa*.

S1j) Brown howler monkey *Alouatta guariba*.

Note these photographs of whole seeds do not show small but key details such as the small teeth marks made by mice, or the marks made by the lower mandible of parrots on the opposite side of the seed.

Photographs taken by J. Martinez (a,b) and F. Hiraldo (c-j).

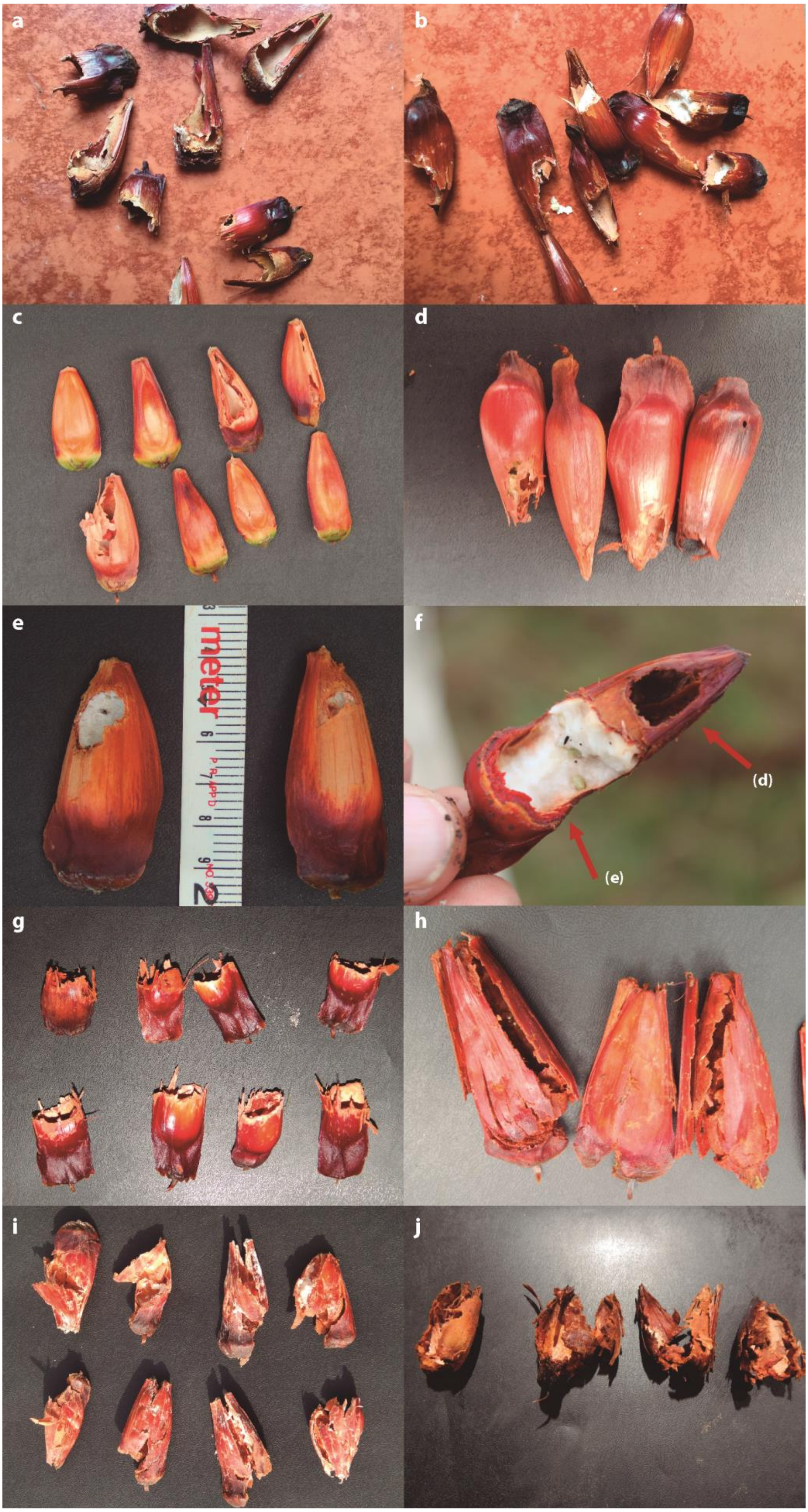
a
b
c
d
e
meter
f
(d)
(e)
g
h
i
j
